# Two decades of harnessing standing genetic variation for physiological traits to improve drought tolerance in maize

**DOI:** 10.1093/jxb/erad231

**Published:** 2023-06-24

**Authors:** Carlos D Messina, Carla Gho, Graeme L Hammer, Tom Tang, Mark Cooper

**Affiliations:** Horticultural Sciences Department, University of Florida, Gainesville, FL, USA; ARC Centre of Excellence for Plant Success in Nature and Agriculture, The University of Queensland, Brisbane, Qld 4072, Australia; School of Agriculture & Food Sciences, The University of Queensland, Brisbane, Qld 4072, Australia; ARC Centre of Excellence for Plant Success in Nature and Agriculture, The University of Queensland, Brisbane, Qld 4072, Australia; Queensland Alliance for Agriculture and Food Innovation, The University of Queensland, Brisbane, Qld 4072, Australia; Corteva Agrisciences, Johnston, IA, USA; ARC Centre of Excellence for Plant Success in Nature and Agriculture, The University of Queensland, Brisbane, Qld 4072, Australia; Queensland Alliance for Agriculture and Food Innovation, The University of Queensland, Brisbane, Qld 4072, Australia; IRD - Institut de Recherche pour le Développement, France

**Keywords:** Crop growth models, drought tolerance, genomic selection, maize, plant breeding, standing genetic variation, Shannon information theory

## Abstract

We review approaches to maize breeding for improved drought tolerance during flowering and grain filling in the central and western US corn belt and place our findings in the context of results from public breeding. Here we show that after two decades of dedicated breeding efforts, the rate of crop improvement under drought increased from 6.2 g m^−2^ year^−1^ to 7.5 g m^−2^ year^−1^, closing the genetic gain gap with respect to the 8.6 g m^−2^ year^–1^ observed under water-sufficient conditions. The improvement relative to the long-term genetic gain was possible by harnessing favourable alleles for physiological traits available in the reference population of genotypes. Experimentation in managed stress environments that maximized the genetic correlation with target environments was key for breeders to identify and select for these alleles. We also show that the embedding of physiological understanding within genomic selection methods via crop growth models can hasten genetic gain under drought. We estimate a prediction accuracy differential (Δ*r*) above current prediction approaches of ~30% (Δ*r*=0.11, *r*=0.38), which increases with increasing complexity of the trait environment system as estimated by Shannon information theory. We propose this framework to inform breeding strategies for drought stress across geographies and crops.

## Teaser

Harnessing managed environments, genomic selection, and crop models enabled utilization of genetic variation for physiological traits available within elite germplasm of a long-term commercial breeding program to improve drought tolerance.

## Introduction

### Planetary water resources for agriculture under increasing stress

Crop distribution and productivity from the regional to the global scale is determined by the amount and pattern of water availability in the absence of temperature limitations (Bunting *et al.*, 1982). In a warming and drier global climate, crop redistribution should be anticipated. Drought episodes will increase with increasing climate variability due to reduced precipitation in mid-to-low latitudes and runoff and infiltration in high latitudes (Rosegrant *et al.*, 2009). Agricultural drought is exacerbated because the loss of topsoil to erosion leads to lower soil water availability for use by crops. It is estimated that 35% of the loss of agricultural area is related to loss of the soil A horizon (Montgomery, 2007; Thaler *et al.*, 2021, 2022), and the degradation continues at a rate of 1 mm year^–1^ in the USA alone. At a global scale, drought in dryland agriculture increased ~1% over the period 1961–2013 (IPCC, 2019). Water demand is increasing and stressing groundwater aquifers, which are mainly located in the tropics and subtropics and in populated agricultural areas (Richey *et al.*, 2015; Rodell *et al.*, 2018). Regeneration of water resources in aquifers while closing productivity gaps through enhanced agricultural technology in rainfed and irrigated agriculture is imperative for sustainable production and food security (Antle and Ray, 2020). However, geographical differences in the repeatability, type, and frequency of occurrence of drought types create challenges to breeders and agronomists that vary in magnitude and complexity. For example, water deficits in major production areas of the world occur around flowering time and grain filling (Harrison *et al.*, 2014; Messina *et al.*, 2015; Marengo *et al.*, 2021). These patterns are repeatable and are well defined, enabling breeders clear objectives. In contrast, drought stress environments in Africa are complex, which stems from the interplay between abiotic (onset, duration, and within-season pattern of rainfall; maximum temperature; subsoil pH) and socioeconomic factors all contributing to create a mosaic of environment types that complicate breeding decisions and hamper genetic progress (Bänziger *et al*., 2006).

## Long-term crop improvement is the foundation for drought breeding: closing yield gaps

**Fig. 1.**
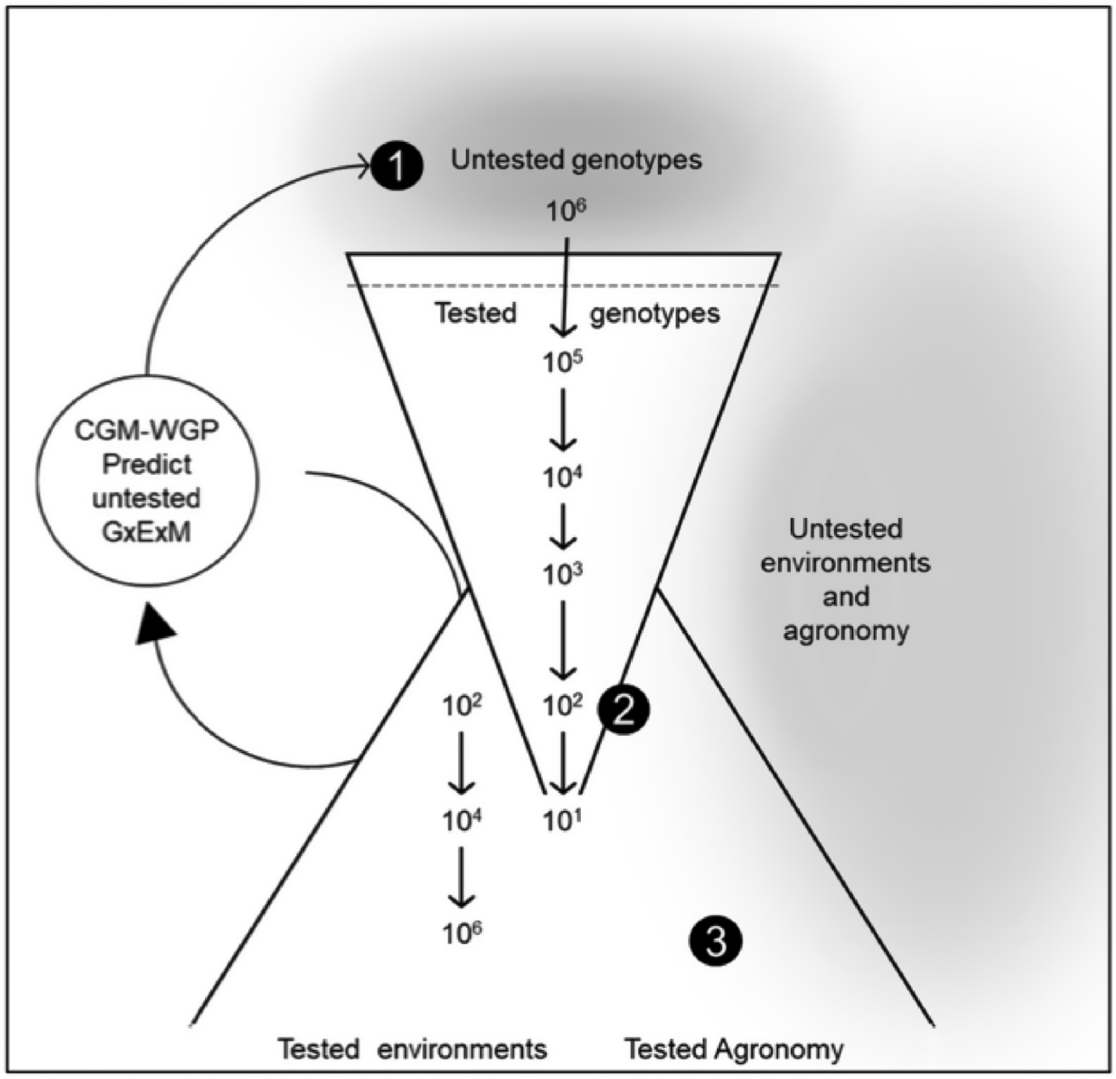
Schematic of a product development process pipeline in a seed industry from the creating of genotypes that were never tested in the field (1) to the time these are tested at scale (2) to the optimization of agronomic practices and growth in farmers’ fields (3). Crop growth model–whole genome prediction (CGM-WGP) methodology uses statistical learning and biological understanding to enable prediction and it is built iteratively as more information is gained through the process development pipeline.

Plant breeding is the single most impactful agricultural technology, contributing to increasing yield potential and to closing yield gaps in maize across rainfed and irrigated systems worldwide. The impact of maize breeding in the USA (Duvick *et al*., 2004; Gaffney *et al.* 2015; McFadden *et al*., 2019) and Africa within the CGIAR system (Bänziger *et al.*, 2006; Prasanna *et al.*, 2021; Krishna *et al.*, 2023) is testament of the extraordinary impact of plant breeding on farmers’ livelihoods. The objective of commercial breeding programs is to make the highest possible rate of genetic gain for one or more traits at the minimum cost (Cooper *et al*., 2014b; Ramirez‐Villegas *et al.*, 2020). Breeding objectives for maize in the US corn belt generally include yield improvement in rainfed and irrigated systems, drought tolerance, disease tolerance, and incorporation of transgenic traits for insect and herbicide resistance, among others. A general structure of a breeding program and the hybrid development pipeline is described in Cooper *et al.* (2014b). Briefly, in hybrid crops such as maize, a breeding program is the result of running two breeding programs (female and male programs) in parallel that complement each other (Fig. 1). Traits conferring drought tolerance may be contributed from lines identified in the female or male heterotic group, or as the result of heterosis (Betrán *et al.*, 2003; Barker *et al.*, 2005; van Eeuwijk *et al.*, 2010). At the industrial scale, a drought breeding program can be centralized or distributed. In the latter case, the program is the result of a collective making independent, yet interconnected decisions based on shared germplasm resources and the exchange of elite lines (see Cooper *et al*., 2014b). In this case, adaptive traits could be contributed from any of the active programs for temperate maize (Cooper *et al.*, 2014b; Technow *et al*., 2021). Once superior maize lines are identified for general combining ability, further testing occurs to identify lines with superior specific combining ability in hybrid combinations. During the testing and advancement of hybrids, one or more transgenes are introgressed into the parental lines of the hybrids prior to their commercial release; most if not all transgenes in active use in breeding confer herbicide resistance and protection against insects (McFadden *et al*., 2019). Throughout various stages of testing and selection, the number of individuals tested in field trials reduces to tens of hybrids. Prior to commercialization, these hybrids are evaluated in large areas in thousands of locations (Fig. 1; Gaffney *et al.*, 2015). Around the time of commercialization, agronomists undertake additional research to identify management practices for optimal hybrid performance in farmers’ fields. Further knowledge about the product is gained once the hybrids are grown in farmers’ fields (Fig. 1). At the early stages of breeding, genotypes are evaluated in few environments, with the number of test environments growing exponentially as these hybrids move through the pipeline. It is not until advanced stages of product evaluation that the hybrid norms of reaction and responses to agronomic management are understood (Cooper and Messina, 2023).

The application of this process and reciprocal recurrent selection over almost 100 years of breeding resulted in increased temperate maize yields at an average rate of 8.6 g m^–2^ year^–2^ (Cooper *et al*., 2014b; Messina *et al*., 2022b). Because the target population of environments (TPE) in the US corn belt includes various water deficit types (flowering stress, grain filling stress, terminal drought; Campos *et al.*, 2004; Barker *et al.*, 2005; Löffler *et al*., 2005; Messina *et al.*, 2015), breeders also improved yields under water deficit at a rate of 6.2 g m^–2^ year^–1^ (Cooper *et al*., 2014b). Crop improvement was largely driven by phenotypic selection, with molecular breeding methods contributing to maintain or increase the rate of genetic gain in the first part of the 21st century. Realized long-term genetic gain for yield occurred despite the many changes in agronomy practices, the effects of climate change (IPCC, 2019), and environmental degradation (Montgomery, 2007; Thaler *et al.*, 2021, 2022). Genetic improvement enabled the intensification of agronomic practices, such as early planting and increased plant population, and proved synergistic (Duvick *et al*., 2004). However, genetic gain was not dependent on any individual technology: agronomic such as irrigation, pesticides, and fertilizers; biological such as transgenes; or breeding such as markers and double haploids.

## Dedicated drought breeding closes the genetic gain gap

Over the last decade, society witnessed the largest expansion of agricultural land planted with drought-tolerant maize (*Zea mays* L.) After the widespread drought event in 2012 (Boyer *et al.*, 2013), the average area of the US corn belt planted to drought-tolerant maize hybrids grew quickly to >20% of the total area (McFadden *et al*., 2019). In drought-prone areas in the western US corn belt, the land allocated to drought-tolerant maize can reach 40–60%, as documented for the states of Nebraska and Kansas. While maize hybrids characterized for superior tolerance to water deficits in the US corn belt were commercialized over 50 years of breeding, since the implementation of drought trials in the 1950s it was recognized that there was an important need to accelerate breeding for drought tolerance (Campos *et al.*, 2004; Barker *et al.*, 2005). The differential genetic gain between water-sufficient and water-limited environments was identified in genetic gain studies (Cooper *et al*., 2014b) and at regional scale in the collective farmers’ fields (Lobell *et al*., 2014). Dedicated research efforts emerged that resulted in the commercialization of AQUAmax® hybrids (AQ hereafter) in 2011 and underpinned the change in land dedicated to cultivating tolerant maize.

AQ maize is the most studied brand of maize of this class, and thus is the focus of this research. Over thousands of comparisons and environments in contrasting geographies, AQ maize yielded 37 g m^–2^ more than non-AQ maize when exposed to drought stress. Yield improvement under drought increased with planting density from 4.7 plants to at least 6.9 plants m^–2^, where the yield difference was 50 g m^–2^ (Gaffney *et al.*, 2015). An important attribute of AQ hybrids is that the yield improvement under water deficit did not come at the expense of reduced performance under irrigation and high rainfall water-sufficient environments (Hao *et al*., 2015a, b; Adee *et al*., 2016; Gaffney *et al*., 2015; Lindsey and Thomison, 2016; Zhao *et al.*, 2018; Messina *et al*., 2022b). This outcome of breeding is consistent with well-defined objectives. AQ technology was developed for current cropping systems, but increased seeding rates were required for these hybrids to fully express their biological potential (Gaffney *et al.*, 2015; Lindsey and Thomison, 2016). On-farm trials followed to demonstrate the synergisms between improved genotypes and agronomic management, which takes the form of an integrated genotype×management (G×M) technology (Gaffney *et al.*, 2015).

The onset of efforts to improve drought tolerance has created an unreplicated experiment (breeding for improved drought tolerance versus business-as-usual industry breeding) that enabled the comparison of responses to selection for yield under water-sufficient and limited conditions. This divergence in selection objectives gives a unique opportunity to study changes and the impact of technologies conducive to closing the genetic gain gap. After two decades, we can predict using the AQ drought breeding experiment that it is possible to accelerate genetic gain in maize whenever the breeding program builds upon long-term efforts. This result can probably apply to other crops and geographies. After two decades of dedicated breeding and technology development and deployment since the late 2000s, the rate of crop improvement under drought increased from 5.6 g m^−2^ year^−1^ to 7.5 g m^−2^ year^−1^, closing the genetic gain gap with respect to the 8.5 g m^−2^ year^−1^ observed under water-sufficient conditions (Messina *et al*., 2022b). In agreement with prior studies, the newest cohort of AQ hybrids are more resilient to stress at higher plant populations (Messina *et al*., 2022b). The application of this development approach enabled the replication of the results for the drought-prone environments (see Marengo *et al.*, 2021) of Safrinha systems in Brazil (Nurmberg *et al.*, 2022).

While molecular breeding made feasible the development of most commercial drought-tolerant hybrids (Cooper *et al*., 2014a, b), gene editing and transgenic approaches demonstrated the potential for further yield improvement under water deficit (Castiglioni *et al.*, 2008; Guo *et al.*, 2014; Habben *et al.*, 2014; Nuccio *et al*., 2015; Shi *et al.*, 2015, 2017; Adee *et al.*, 2016; Schussler *et al.*, 2022). A retrospective account of the development of drought-tolerant maize would be incomplete by not mentioning fundamental research that can contribute to improve drought tolerance beyond the current levels measured in elite germplasm. For example, gene-edited maize for modified expression of the *ARGOS8* gene yielded 33 g m^–2^ more than a control under flowering stress but not grain fill stress (Shi *et al.*, 2017). Similarly, under water deficit, maize transformed with *ARGOS8* yielded 35 g m^–2^ more than transgene negative hybrids (Shi *et al.*, 2015). Maize transformed with trehalose-6-phosphate (T6P) from rice showed yield improvement under water deficits >9% (Nuccio *et al*., 2015). Overexpression of *zmm28* in several lines was shown to increase yields of hybrids by >4% on average under water deficit conditions (Schussler *et al.*, 2022).

## Harnessing standing genetic variation for physiological traits

The crop genetic improvement for drought tolerance relative to the long-term genetic gain (Cooper *et al*., 2014a; Messina *et al*., 2022a) was possible by harnessing favorable alleles for physiological traits available within the elite germplasm of the commercial reference population of genotypes (RPG). The RPG is the active mixture of extant genotypes being used by a breeding program that was derived through combinations of selection and intermating processes that contain the extant combinations of founder haplotypes. Three core technologies, managed stress environments (MSEs), precision phenotyping, and genome-to-phenome modeling, enabled the use of the standing genetic variation, founder alleles at a given locus present in the target population of genotypes, within breeding programs.

MSEs refer to the collective of experimental sites where controlled (managed) perturbations can be introduced into the crop management system to expose genetic variation for traits that contribute to drought adaptation (Fischer *et al.*, 1989; Bänziger and Cooper, 2001; Rebetzke *et al.*, 2013; Cooper *et al*., 2014a). Precision phenotyping, most often used in MSEs, refers to the set of tools that could be used to measure the state of the crop system and the rates of physiological processes (Sinclair, 2011; Araus and Cairns, 2014; Cooper *et al*., 2014b; Araus *et al.*, 2018; Reynolds *et al.*, 2020). Enviromics can be defined in a similar manner when we refer to the crop–environment system (Chapman *et al.*, 2003; Cooper and Messina, 2021). Genome-to-phenome models are mathematical formulations and cognitive representations of knowledge useful to predict one or more crop phenotypes from genetic and genomic information (Meuwissen *et al.*, 2001; Bernardo and Yu, 2007; Jarquin *et al*., 2014; Cooper and Messina, 2023).

To relate these technologies to their contribution to genetic gain, we use a formulation of the breeder’s equation (Cooper and DeLacy 1994; Podlich *et al.* 1999; Annicchiarico *et al.*, 2015; Cooper *et al*., 2023b) that predicts the rate of gain [∆*G*_(MET,TPE)_] in the TPE using data collected in multiple environment trials (MET),


$$\begin{array}{l} \Delta G_{\left(\text{MET,TPE}\right)}=i_{\left(\text{MET}\right)}\times {r_{a}}_{\left(\text{MET}\right)}\times {r_{a}}_{\left(\text{MET},\text{TPE}\right)}\times {\sigma_{a}}_{\left(\text{TPE}\right)} \end{array}$$ (1)

where *i*_(MET)_ is the standardized selection differential informed by the analyses of data collected in the MET, *r*_*a*(MET)_ is the prediction accuracy for the trait(s) of interest estimated using the marker, phenotype, and environmental information content in the MET training datasets, and the prediction algorithm, *r*_*a*(MET,TPE)_, is the genetic correlation between the additive genetic effects estimated using the MET training data sets and the additive genetic effects for the physiological and end of season trait targets in the TPE. The term σ_*a*(TPE)_ is the additive genetic variation for the traits within the TPE, not the MET as in prior formulations of the breeder’s equation.

The concerted application of these technologies enabled the design of evaluation and prediction systems relevant for the germplasm that both expose variation for adaptive traits and maximize the genetic correlation between MET and TPE. Estimated *r*_*a*(MET,TPE)_ for temperate maize varied between 0.41 and 0.70 (Cooper *et al*., 2014a) and for tropical maize in Africa environments it was 0.58 (Menkir *et al.*, 2022). The positive rates of genetic gain in performance in environments created in MSEs that resemble and stratify the main environment challenges of the TPE suggest positive values for *r*_*a*(MET,TPE)_ for tropical maize in Latin America (Bänziger and Cooper, 2001) and Africa (Bänziger *et al*., 2006). Agronomic practices including precision planting and irrigation were required to achieve *r*_*a*(MET,TPE)_ values that proved useful for selection for improved drought tolerance that was realized in the TPE.

## Managed stress environments enabled convergent research and breeding

**Fig. 2.**
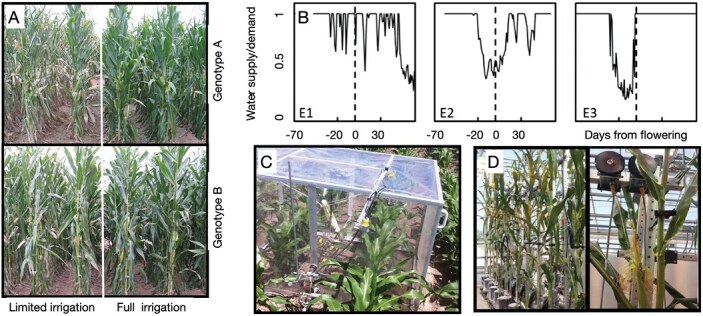
Examples of breeding technologies to evaluate maize germplasm in field conditions (A), understand drought environments (B), and validate traits in controlled environments for gas exchange response to vapor pressure deficit (C), and silk elongation response to soil water (D).

MSEs enabled research with the objectives of improving the understanding of the physiological underpinnings of drought tolerance, mapping the genetic architecture of adaptive traits, conducting environmental characterizations, implementing environmics within breeding programs, and improving agronomic practice (Bolaños and Edmeades 1996; Monneveux *et al*., 2006; Cooper *et al*., 2014a, b; Fig. 2). The implementation of drip tape systems was a pivotal technological change that enabled precise recording and control of the amount and timing of watering. Detailed record keeping made enviromics through soil sensing, automated weather stations, and crop modeling possible (Cooper and Messina, 2021). Figure 2A shows an example for how precision management in an MSE can create the conditions to discriminate hybrids for adaptive traits and yield. Environmental modeling calculates the ratio of water supply from the soil and the water demand as determined by growth stage, soil properties, and atmospheric conditions (Cooper and Messina, 2021). In Figure 2B we show three examples that help understand how the irrigation and agronomic management helps to precisely expose crops to conditions conducive to discriminate germplasm for improved performance under stress imposed during critical reproductive periods (Fig. 2B, E1, grain fill; E2, silking and kernel abortion; E3, silk elongation).

Imposing drought at flowering time enabled exposing genetic variation for barrenness and protogyny (negative difference between anthesis and silking), a trait that was associated with drought tolerance across germplasm pools (Bolaños and Edmeades, 1993, 1996; Cooper *et al*., 2014a). Silk elongation is highly sensitive to water deficit (Hall *et al.*, 1982; Fuad Hassan *et al.*, 2008). Synchronous silk development was implicated in improved kernel set due to reduced apical kernel abortion under water deficit (Oury *et al.*, 2015). Water deficits imposed just after silk emergence enable separating germplasm for tolerance to kernel abortion, barrenness, and yield due to carbon starvation during the lag phase (Edmeades *et al*., 1993; Monneveux *et al.*, 2006; Messina *et al.*, 2021).

Water stress imposed during grain fill to crops growing in soil with high soil water-holding capacity and no restrictions to root growth enabled breeders to identify germplasm for improved water capture and/or improved effectives of the root system to maintain water flow for a given the leaf area of the plant (van Oosterom *et al.*, 2016). However, selection for improved water capture and root growth *per se* was elusive for crop improvement in both temperate (Reyes *et al.*, 2015; Messina *et al*., 2021) and tropical (Bolaños *et al.*, 1993) maize germplasm. In temperate germplasm, the increased plant population was conducive to higher water uptake (Messina *et al*., 2021) rather than the changes in root architecture at the plant level. The control of water uptake take place at the crop rather than the plant level.

Imposing drought stress during grain filling including the lag phase in soils with high soil water-holding capacity but with restrictions to root growth below 1–1.2 m, enabled breeders to identify contrasting germplasm for traits such as limited transpiration (Choudhary *et al.*, 2013; Shekoofa *et al.*, 2015; Tardieu *et al.*, 2017), canopy expansion (Lacube *et al.*, 2017), and silk elongation response to water deficit (Cooper *et al*., 2014a; Fuad-Hassan, 2008; Messina *et al.*, 2019). Leaf and silk elongation responses to water deficit were validated in controlled environments (Fig. 2C; Turc *et al.*, 2016), while transpiration response to vapor pressure deficit was validated at field scale using chambers to measure gas exchange in small canopies (Fig. 2D).

These biological insights and technical developments were used to design experimental management strategies in key environments to expose genetic variation for adaptive traits. For example, MSEs were implemented in Woodland, CA and Viluco, Chile to expose the germplasm to conditions that were conducive to expressing variation for traits that affect the dynamics of the water balance, water capture, and reproductive resilience. The application of the robust quantitative genetic framework (Lynch and Walsh, 1998; Walsh and Lynch, 2018), biological knowledge, and precision phenotyping in these MSEs led to the observed large impact on genetic gain (Gaffney *et al.*, 2015; Messina *et al*., 2022b; Cooper and Messina, 2023). This finding for the US corn belt breeding can apply to similar programs seeking to improve drought tolerance in Europe, Brazil, and parts of Africa (4; Harrison *et al.*, 2014; Marengo *et al.*, 2021).

## Multiple linked bouts of crop adaptation to drought stress in the US corn belt

Breeding is a search process for creating genotypes with higher adaptation to agricultural drought; the crop needs to survive but also produce economic yield. Radical changes in the search for new combinations of alleles and physiological traits can lead to maladapted germplasm that cannot regain competitiveness. Large programs operate in a way that resembles both the Fisher geometrical model and Wright rugged landscapes (Orr, 2005). The use of multiple breeding programs operating in parallel enables exploration of a multi-peaked performance landscape (Messina *et al*., 2011) that emerges from the genetic architecture of traits as defined by the number of genes and their interaction (Cooper *et al*., 2005), with each program taking small steps to increase performance within a peak as proposed by the geometrical model (Cooper *et al*., 2014b; Technow *et al.*, 2021). Breeding objectives can be shared or not among parallel breeding programs. In the AQ program, the shared objective was to increase drought tolerance while maintaining parity performance under water-sufficient conditions. Specific objectives for the various programs are often related to standability, plant height, and disease tolerance, among others. In drought breeding, this process has another layer of complexity as improved germplasm opens up opportunities to change the agronomic practices used to manage water availability. In other words, the performance landscape is like a wave that changes shape with changes in agronomic management. Long-term crop improvement in maize was underpinned by genetic improvement for stress tolerance followed by a change in agronomic practices such as plant population and early plantings (Duvick *et al*., 2004). This pattern was conserved and actively utilized in drought breeding (Gaffney *et al.*, 2015; Messina *et al*., 2022b).

Drought breeding led to the family of AQ hybrids that can maintain harvest index (HI) under water deficit (Hao *et al*., 2015b; Mounce *et al.*, 2016; Zhao *et al.*, 2018), and can withstand higher than normal plant populations (Irmak *et al.* 2020; Messina *et al*., 2022a). This combination enables the crop to fully utilize the available soil water. In addition to a plausible increase in water capture due to the ability to withstand higher than normal stands under water-sufficient conditions, modeling studies using AQ hybrids indicated that reduced stomatal conductance under high vapor pressure deficit (Messina *et al.*, 2015) can increase the water use during the reproductive period at the expense of the vegetative phase in drought stress environments. The improved water status during the critical window for kernel set, and the smaller size of the ear at silking (Messina *et al.*, 2011, 2018), can underpin the observed protogyny and shortened anthesis–silking interval (ASI; Cooper *et al*., 2014a, b), higher silk number under water deficit (Cooper *et al*., 2014a; Messina *et al.*, 2019), and the maintenance of HI under drought. Under grain filling stress, genetic gain was found to decrease when the population increased beyond 7.5 plants m^–2^ (Messina *et al*., 2022a). This optimum indicates that the observed increase in reproductive resilience under flowering stress treatments extended to grain fill, probably through reduced abortion, but also that limited water availability may have led to an early termination of grain fill, limiting the realization of an increased kernel set. We have observed that some hybrids within the AQ set restrict the number of synchronous emergences of silks, which in turn restricts cob growth and the competition for resources between fertilized ovules and the cob. Results from experimentation (Shen *et al*., 2020) and simulation suggest the hypothesis that competition for resources between cohorts of kernels and the cob during the lag phase underpins the determination of kernel number, and that source/sink rebalancing due to fertilization failure regulates carbon allocation between the cob and the growing kernels, and thus kernel set under stress (Messina *et al.*, 2019). Prior studies suggest that yield improvement was not associated with increased water capture at constant density (Reyes *et al.*, 2015; Messina *et al.*, 2021) and that AQ hybrids, at least from the first generations, rather shifted the patterns of water use instead of increasing total water capture (Cooper *et al*., 2014a; Messina *et al.*, 2015). The absence of a differential genetic gain under well-watered conditions is consistent with the selection criteria focused on improvement of yield under water deficit while not compromising yield potential under well-watered conditions (Gaffney *et al.*, 2015; Messina *et al*., 2022a). Taken together, the evidence suggests that drought breeding is selecting for multiple mechanisms of drought tolerance, and the combination of these mechanisms may underpin the sustained genetic gain over the two decades of crop improvement (Campos *et al.*, 2004; Barker *et al.*, 2005; Gaffney *et al.*, 2015; Messina *et al*., 2022a). While increasing water capture through improved root growth and/or root system efficiency was advocated as a path to improve drought tolerance in maize (Tuberosa *et al.*, 2002; Hammer *et al.*, 2009; Ruta *et al.*, 2010; Messina *et al.*, 2011; van Oosterom *et al.*, 2016; Hochholdinger *et al.*, 2018), there is no strong evidence that suggests that this path was fully exploited by current hybrids, and thus remains an unexplored opportunity (Diepenbrock *et al.* 2022).

## Genome-to-phenome models increase prediction accuracy for drought environments

The application of gene-to-phenotype prediction methodologies, mainly genomic selection (GS), enabled the revolution in molecular breeding (Meuwissen *et al.*, 2001; Bernardo and Yu, 2007; Gianola *et al.*, 2009; Cooper *et al*., 2014b; Heslot *et al.*, 2014; Crossa *et al.*, 2017). This transformation in breeding was only possible because of the convergence of molecular approaches with other technologies such as double haploid production, and precision phenotyping (Cooper *et al*., 2014b). These technologies are applied routinely at early stages of breeding programs to enable the generation of, and selection upon, large numbers of untested and tested individuals, increasing the size of the breeding programs (Fig. 1; Araus *et al.*, 2018; Hammer *et al.*, 2019; Washburn *et al.*, 2020). However, ubiquitous genotype×environment (G×E) interactions under water-limited conditions place a cap on the rate of attainable genetic gain (Cooper *et al*., 2020a, 2023b; Cooper and Messina, 2023).

Transdisciplinary approaches that leverage biological insights, and statistical learning methods are changing the ways in which we approach crop improvement (Hammer *et al.*, 2019; Messina *et al*., 2020b). The challenge to prediction that stems from the need to predict G×E×M interactions motivated modeling of G×E within statistical frameworks (Boer *et al.*, 2007; Jarquin *et al*., 2014, 2017, 2020; Li *et al.*, 2018; Millet *et al.*, 2019; Rincent *et al.*, 2019; de los Campos *et al.*, 2020). Although these statistical approaches are essentially static in character, they can capture the dynamics of crop systems when biological understanding is leveraged in the selection of environmental covariates and the aggregation of information by stages of development known to be of critical importance for yield determination (Bustos-Korts *et al*., 2019a, b; Millet *et al*., 2019). Other approaches fully incorporate the dynamics of the crop system. The integration of GS with crop growth models (CGM–GS, Technow *et al*., 2015; Cooper *et al*., 2016; Messina *et al*., 2018, 2022b) is such an example. Other examples were advocated by linking quantitative trait loci (QTLs) with crop models (Yin *et al*., 2004). The central hypothesis underlying CGM–GS is that by harnessing biophysical knowledge through the CGM to capture the gene-to-phenotype relationships for traits contributing to yield variation and consequently trait×environment interactions, it is possible to (i) understand effects of allele substitution and genetic variation for traits across environments, and (ii) increase predictive skill for end point traits such as yield, yield stability, and yield norms-of-reaction (Messina *et al*., 2022a, b). Within this framework, the CGM acts as a link function between genotype and phenotype (Cooper *et al*., 2020b). Trained CGM can predict phenotypes for a given genotype and management for productivity and water use, nitrogen loss, and other metrics that can enable decision makers to assess the value of genotypes in the context of environment sustainability (Peng *et al.*, 2020; Messina *et al*., 2022b; Cooper and Messina, 2023). A multi-dimensional framework to inform selection decisions can evaluate genotypes for productivity at a given level of evapotranspiration, the range of which is defined by the TPE (Peng *et al.*, 2020; Messina *et al*., 2022b).

Because physiological traits in CGM–GS are directly modeled using marker information, it is possible to estimate these with accuracies that are dependent on the degree of relatedness between the training populations, used to generate prior knowledge, and the genotypes of interest. Physiological traits are parameters in the crop model that quantify, for example, how transpiration is converted to mass (Tanner and Sinclair, 1983). The stringency of experimental designs and information management required to use CGM–GS increases but the field experimentation demands decreases because of the increase in the information content resulting from experimentation. In CGM–GS, it is not necessary to measure any physiological traits. However, it is critical to expose the germplasm to environments that elicit trait×environment interactions to enable the estimation of parameters (Messina *et al.*, 2018). The use of MSEs (Fig. 2) enables research for direct observation of trait physiology or to elicit germplasm response to drought and expose genetic variation for adaptive traits contributing to yield variation. Whether some traits are measured or estimated, CGM–GS enables breeders to access biological knowledge, physiological and genetic, to inform selection decisions at early stages of breeding when phenotyping of physiological traits is limited at an industrial scale (Diepenbrock *et al.*, 2022). Advances in high-throughput phenomics (Araus and Cairns, 2014; Araus *et al.*, 2018; Reynolds *et al.*, 2020), our understanding of how trait and state phenotypes are connected within modelling frameworks (Jones *et al.*, 2017; Soufizadeh *et al.*, 2018; van Eeuwijk *et al.*, 2019; Wu *et al.*, 2019), and the possibility to assimilate phenomics and genomics information within CGM–GS (Messina *et al.*, 2018) will increase our understanding of adaptation to drought and predictability thereof.

**Fig. 3.**
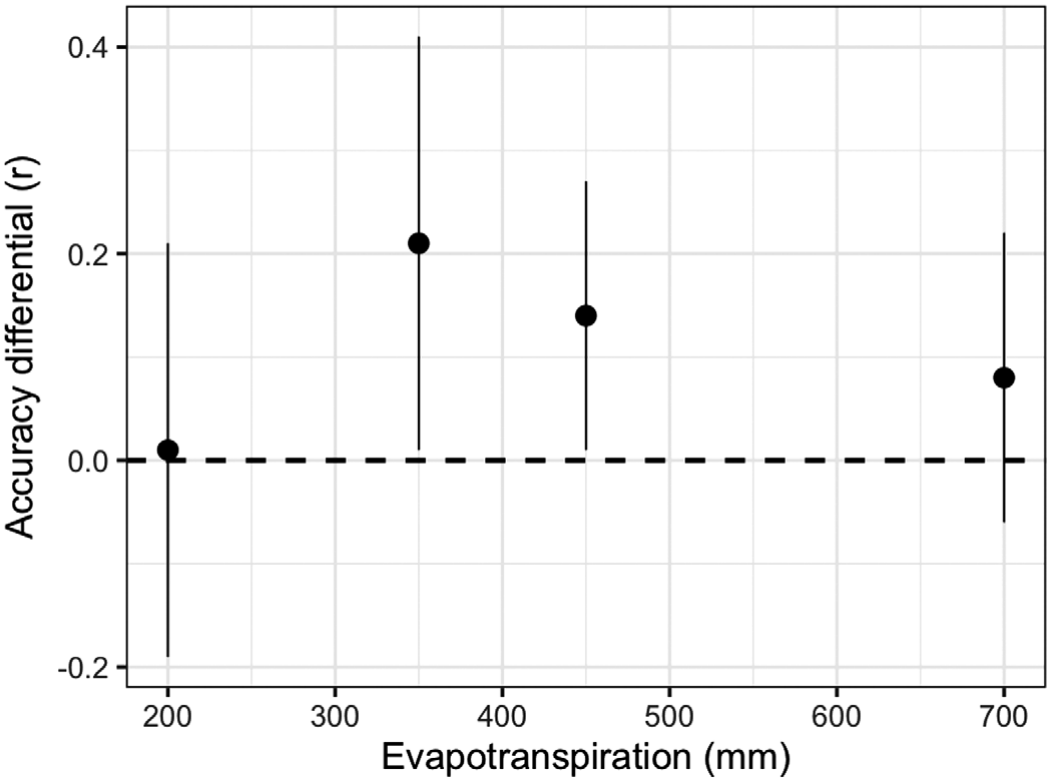
Average prediction accuracy differential between the crop growth model—genomic selection methodology and Bayes A method versus level of evapotranspiration [data from studies by Diepenbrock *et al.* (2022) and Messina *et al*. (2022b)]. Accuracy estimated by the correlation coefficient (*r*) for the out of the sample validation set.

Results from Cooper *et al*. (2016) demonstrated empirical application of CGM–GS for a drought study where there was little improvement over genomic best linear unbiased prediction (BLUP) alone. The drought environments considered by Cooper *et al*. (2016) discriminated the germplasm in a very similar manner. There was a high genetic correlation (*r*_G_=0.88) for yield between the two flowering stress environments included in their study. While the timing of water deficit varied between the two treatments, the same physiological mechanism underpinned the observed tolerance to drought, limiting the expression of differential G×E for yield (Cooper *et al*., 2016). In contrast, significant improvements in predictive skill of CGM–GS over GS alone were observed when contrasting environments (deficit irrigation and full irrigation) and populations expressing contrasting genetic correlations (*r*_G_ =–0.08 to 0.49) were considered (Messina *et al.*, 2018). Further studies including large populations and multiple environments confirmed this result (Fig. 3; Diepenbrock *et al.* 2022; Messina *et al*., 2022b).

**Fig. 4.**
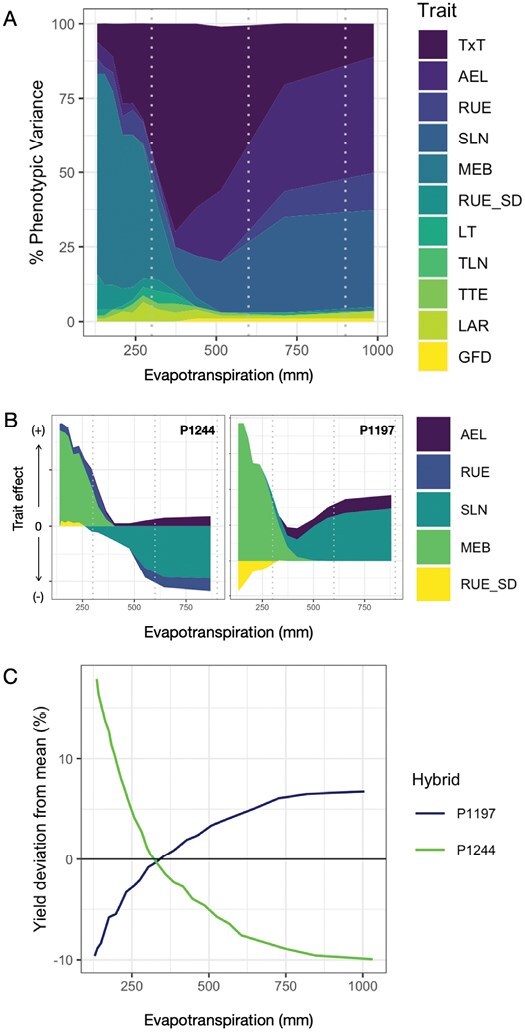
Physiological trait effects on phenotypic variance for yield along an evapotranspiration gradient (A) vary with genotype (effects shown as difference from genotype mean, B) and underpins genotype x environment interactions (C) (Messina *et al*., 2020a). AEL: area of the ear leaf, RUE: radiation use efficiency, SLN: specific leaf nitrogen, MEB: minimum biomass of the ear at silking, RUE_SD: radiation use efficiency response to soil water; LT: transpiration response to vapor pressure deficit, TLN: total leaf number, TTE: thermal time from planting to emergence, LAR: leaf appearance rate, GFD: grain fill duration, TxT: Trait x Trait interactions.

Estimated accuracies of prediction *r*_*a*(MET)_ for models trained with data collected in METs ranged from 0.28 to 0.83 in tropical maize (Crossa *et al*., 2014) and from –0.25 to 0.5 in temperate maize (Cooper *et al*., 2016; Messina *et al*., 2018, 2022b; Diepenbrock *et al*., 2022). The mean predictive skill for the study conducted by Messina *et al*. (2022b) was 0.38 when the largest number of locations were included in the training set. The range of variation was dependent on year and number of locations included in the training set. Combining prior results (Diepenbrock *et al*., 2022; Messina *et al*., 2022b) we show that prediction accuracies are on average greater for CGM–GS than for GS alone (Δ*r*=*r*_CGM-GS_–*r*_GS_= +0.11). This gain in predictive skill is due in part to the ability of CGM–GS to simulate emergent phenotypes and thus predict G×E×M interactions. However, the realization of the prediction accuracy differential (Δ*r*) depends on the environment (Fig. 3), which could be thought of as a function of the complexity of the trait network underpinning crop adaptation. At very low levels of evapotranspiration (<200 mm) and yield (<300 g m^–2^), Δ*r* is low or nil (Fig. 3), because there are few traits underpinning variation for grain yield (Fig. 4A; Messina *et al*., 2020a, Preprint). Silk exertion and prevention of abortion are the major determinants of yield in these low-yielding environments (see trait ear size at silking and its relationship to silk number; Messina *et al*., 2019). As evapotranspiration and yield increase, more traits become involved in the determination of yield variation and, more importantly, the interactions among traits. For yield environments of 1000 g m^–2^, >50% of the phenotypic variance for yield is attributed to interactions among physiological traits (Fig. 4A; Messina *et al*., 2020a, Preprint). The highest Δ*r* is observed at intermediate levels of evapotranspiration for which trait×trait interactions are key drivers of yield (Fig. 3). Under water-sufficient conditions (evapotranspiration=700 mm and yield=1575 g m^–2^; Figs 3, 4A), the trait complexity decreases along with Δ*r*. For these environments, light interception and conversion efficiencies are the main determinants of phenotypic variation in maize (Fig. 4A). In the environmental conditions most typical of humid years in the US corn belt, there is no clear advantage of CGM–GS over GS (Diepenbrock *et al*., 2022). However, under large vapor pressure deficits and water-sufficient conditions, typical of MSEs and dry years in the US corn belt, it is feasible to observe a bifurcation in predictive skill tied to the expression of the limited transpiration trait and the resulting manifestation of lack of correlation in the G×E interactions for yield (Messina *et al*., 2015, 2018). Overall, harnessing biological insights through CGM to enable GS for drought breeding can hasten genetic gain for yield for the mixture of environments of the TPE by increasing *r*_*α*(MET)_ (Equation 1).

## Towards a general predictive breeding framework for drought tolerance

When breeders face emergent phenotypes, such as drought tolerance, which are the result of the interplay between organisms and the environment, integrating biological understanding of trait genetic architecture into trait strategies to predict G×E×M such as crop models proved a practical (Cooper *et al*., 2016) and effective approach to increase the predictability of the system (Diepenbrock *et al.*, 2022; Messina *et al*., 2022b). The improvements in prediction accuracy from using dynamical models were not uniform across levels of evapotranspiration (Diepenbrock *et al.*, 2022; Fig. 3). This observation leads to the hypothesis that the gap in predictability between a static statistical prediction model such as a genomic best linear unbiased prediction (GBLUP; Meuwissen *et al.*, 2001; Bernardo and Yu, 2007) and a dynamical prediction model such as CGM–GS increases with increasing system complexity. The gain in predictive skill increases with the increasing importance of system dynamics for the determination of emergent phenotypes and of these on the expression of G×E×M. In other words, missing heritability and environmentability (de los Campos *et al*., 2020) could be recovered by considering system dynamics within the prediction framework.

**Fig. 5.**
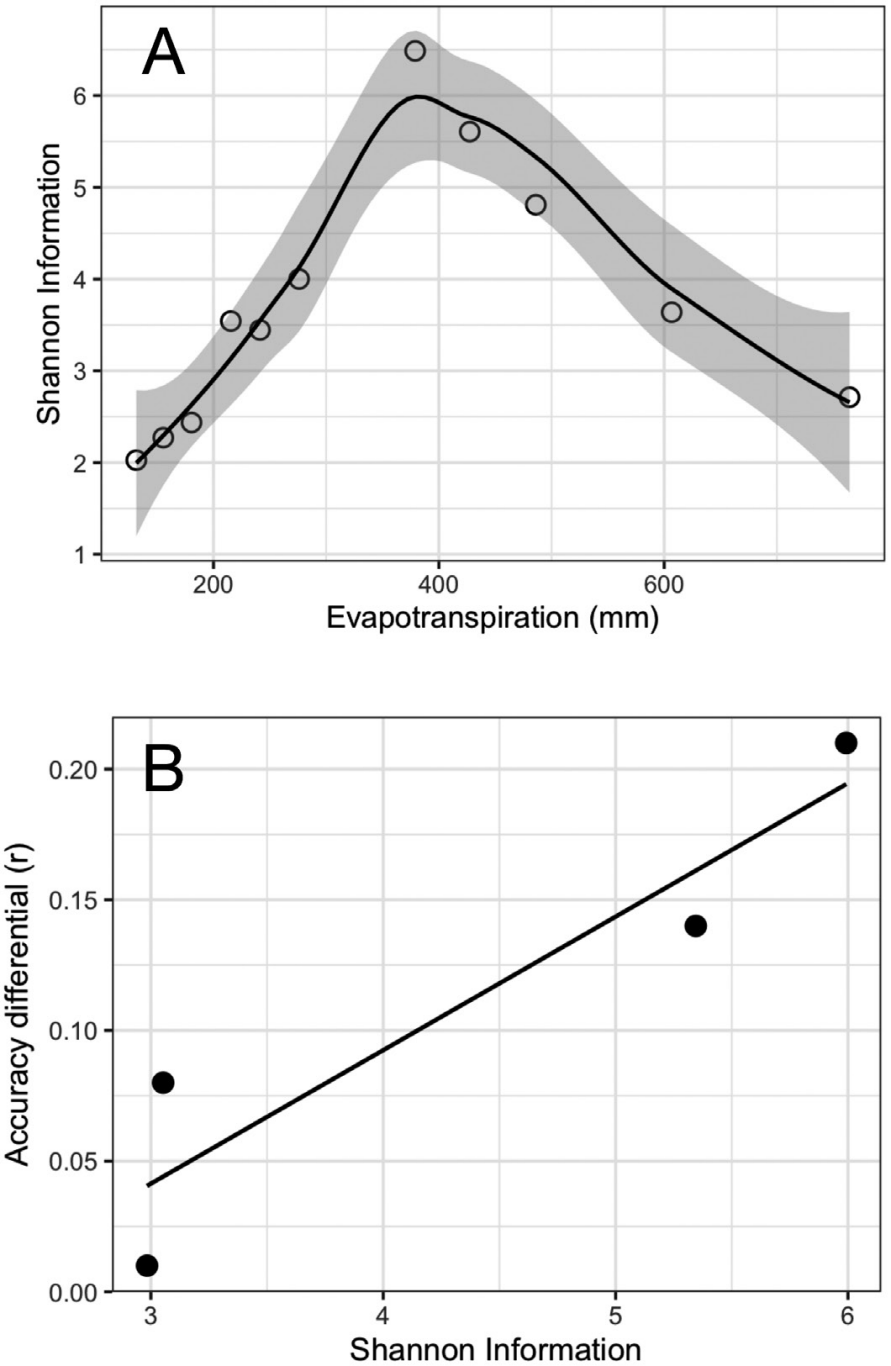
Shannon information theory estimates the variation in complexity of the maize G×M system along a evapotranspiration gradient (A), which suggests that the observed variation in prediction accuracy differential (crop model–whole genome selection versus Bayes A) along the evapotranspiration gradient could be due to expression of complexity in the trait×trait×environment system underpinning yield variation (B).

One way to evaluate this hypothesis, is to use the Shannon information theory (Golan and Harte, 2022),


$$\begin{array}{l} \text{H}=-\underset{\text{T}}{\sum }\;\text{p}\left(\text{T}\right)\times \text{lo}{\text{g}}_{2}\left[\text{p}\left(\text{T}\right)\right] \end{array}$$ (2)

where p(T) is the probability of trait (T) contributing to phenotypic variation of yield at a given level of evapotranspiration. The trait complexity variation relative to the evapotranspiration gradient was estimated from the contribution of nine maize traits to phenotypic variation in a multienvironment trial (Clark *et al*., 2023, Preprint). To account for trait×trait interactions in the calculation of H, we assumed that all possible trait interactions occurred with equal probability. Both the index H (Fig. 5A) and prediction accuracy differential (Fig. 3) followed a non-linear pattern with respect to evapotranspiration that resembled the pattern of the contribution of trait×trait interactions in the explanation of phenotypic variance for yield (Fig. 4A; Clark *et al*., 2023, Preprint). At high or low levels of evapotranspiration, few traits and interactions determine performance differences between genotypes (Clark *et al*., 2023, Preprint; Cooper and Messina, 2023). In contrast, at intermediate levels of evapotranspiration, the pattern of water use over time and trait×trait interaction explain an important fraction of the phenotypic variance. A positive correlation between Shannon information theory and prediction accuracy differential (*r*^2^=0.8; *P*<0.1; df=2) offers evidence for the contribution of CGMs to increase prediction accuracy by acting as link functions that capture the dynamical dependencies among traits and with management and the environment (Cooper *et al.*, 2021; Fig. 5B). This finding provides a first answer to Marjoram’s question about how to improve predictive skills in genetics and genomics: more markers or more biology? (Marjoram *et al.*, 2014). The evidence suggests that there is merit to continuing these investigations with G×E×M systems other than maize in the US corn belt.

A corollary of this finding is that there is an economic and logistics optimum for the application of prediction methodologies in at least drought breeding. Prediction methods encompass a variety of models. Some models are based on additive effects such as GBLUP models and their extensions to the multienvironment case (Boer *et al.*, 2007; Jarquin *et al.*, 2014; Millet *et al*., 2019). Others consider non-additivity because of trait-to-trait interactions and their responses to the environment (van Eeuwijk *et al.*, 2019; Costa-Neto *et al.*, 2021). CGM-based models predict emergent phenotypes as described above, but they demand highly trained human and computational resources (Cooper and Messina, 2023). The relationship between the complexity of the G×E×M system and prediction differential can be used to guide breeders and geneticists on their quest to select a useful modeling strategy. In simple cases, linear models such as QTLs or GBLUP are enough to capture the predictability of the system, for example when the environmental variation is driven by few variables and the trait is regulated by a small number of loci with large effects that depend additively on the environment. The ASI in maize has been successfully predicted with linear-mixed models (e.g. Welcker *et al*., 2007; Cooper *et al*., 2014a). At intermediate levels of complexity, when the environment creates lack of correlation G×E interactions, models based on additive effects that are a linear function of the environmental drivers in a factorial regression model can capture enough system complexity (Jarquin *et al.*, 2014; Millet *et al*., 2019). When complexity is highest, the use of hierarchical dynamic crop growth models is needed, as demonstrated in various studies seeking to improve drought tolerance in maize (Cooper and Messina, 2023).

## Closing yield gaps under drought

**Fig. 6.**
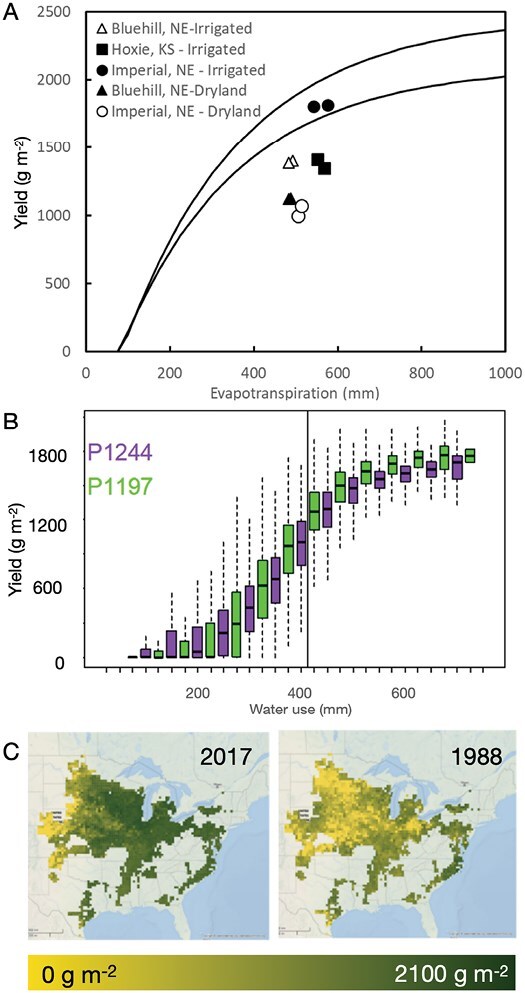
Simulated yields define theoretical maize yield response to evapotranspiration for 80 and 99 percentiles (A, lines), maize yield variability for hybrids with contrasting response to water deficit across a range of evapotranspiration (B), and spatial distribution of yields for 2 years with different drought patterns at 30 × 30 km resolution (C). Yield observations shown in (A) are for a single cross-hybrid grown at three locations in the western US corn belt for maize grown under rainfed and irrigated conditions, and under normal (filled symbols) and increased plant population by 1 plants m^–2^ (open symbols). Data are from Messina *et al*. (2020a).

Improved cultivars enabled agronomic intensification and the efficacy with which crops transform natural resources into biomass and yield outcomes. When the combination of genotype and management is inadequate, yield gaps emerge (Hammer *et al.*, 2014, 2020; Hao *et al.*, 2019; Cooper *et al*., 2020a, 2021, 2023a). Figure 6A shows an example at the farm level, where yields higher than the 80% quantile, an arbitrary measure of efficiency of resource use and conversion (van Ittersum *et al*., 2013), were observed. While different factors, predictable and unpredictable, can underpin these results, they illustrate the opportunity to diagnose and improve decisions on the choice of G and M technology. The use of CGM–GS technology allows the simulation of how genotypes generate yield under various water regimes (Fig. 4B) and what traits can contribute to yield determination along the continuum of low- to high-yield environments (Fig. 4B,C). For example, the hybrid P1244 is more suitable for production in drought-prone environments than P1197 (Fig. 4C). While both hybrids are predicted to have high reproductive resilience to water deficit (Fig. 4B, , Messina *et al.*, 2011, 2019), the hybrid P1244 can recover carbon assimilation better than P1197 after an episode of severe drought (Fig. 4B). The use of CGM–GS thus enables simulation to compare genotype–management combinations anywhere in the US corn belt (Fig. 6C; Cooper *et al*., 2020a) and improve decision making by assessing norms of reaction for each of the hybrids (Fig. 4C). The simulation of means and variability is key to decision making under risk and the implementation of climate-resilient production portfolios that are best suited to the farmers’ risk attitude (Teng *et al.*, 1997; Messina *et al.*, 1999; Jones *et al.*, 2000; Bard and Barry, 2001; Cooper *et al.*, 2021). The characterization of hybrids by their genomic and physiological profiles (Fig. 4B) can further inform the choice of hybrids to reduce the environmental risk to the farmer. Diepenbrock *et al.* (2022) demonstrate how this technology could be applied at the earliest stages of breeding. Further integration with breeding simulation (Podlich and Cooper, 1998; Messina *et al.*, 2011; Gaynor *et al*., 2021) and weighted selection strategies (Podlich *et al.*, 1999) can further accelerate genetic gain for drought by advancing germplasm based on genomics and agronomic management.

## Conclusions and perspectives

Molecular breeding approaches transformed breeding, and dedicated efforts to improve drought tolerance in maize demonstrated sustained genetic gain at the industrial scale and will continue providing the foundation to deliver drought-tolerant maize for the US corn belt. Drought breeding will continue to build upon the system that drives long-term genetic gain. Here, our review demonstrates near-term opportunities to realize yield improvement that may include using technologies that harness both quantitative genetics and physiological frameworks for prediction at early stages of breeding, for placement of hybrids within regions, and design strategies given the drought-tolerant hybrids and agronomic practices available to the farmer. The feasibility to apply technologies to improve drought tolerance in maize from breeding to farm has the potential to accelerate crop improvement by designing and developing improved G×M technologies. Gap analyses enables us to predict the outcome of combining haplotype genetic blocks that control physiological processes and agronomic practices even for genotypes that were created in a breeding program but never tested in the TPE. Gap analyses in a way closes the cycle from breeding to farm and back to breeding.

Prediction methodologies were developed and improved, and demonstrated to have the greatest opportunities to deliver increased rates of crop improvement gain under water deficit conditions. Harnessing biological insights for end-to-end prediction of G×M technologies is a promising path towards increasing crop yields and water productivity. However, there is a clear need for investments in plant science to advance our biological understanding of adaptation, germplasm diversity, algorithm development that improves statistical methodologies, and, of most importance, the development of a new engineering and design paradigm that harnesses complexity science and, by doing so, leverages noise and uncertainty to improve decisions and systems performance. The relationship between Shannon information theory and the predictive accuracy differential provides a framework to guide future research in maize and other crops.

CGM–GS methodology proved to be effective at modeling G×E×M interactions and has potential to improve decisions at all stages of product development and agriculture in drought-prone environments. Further improvements in genetic gain are feasible due to increased *r*_*a*(MET)_ and the design and placement of G×M technologies for the mixture of environments that comprise the TPE, in other words increased *r*_*a*(MET,TPE)_.

While the evaluation of CGM–GS to support maize breeding for the US corn belt was specific to one combination of a statistical and biophysical model, we argue that the results could be generalized to the state that the combination of statistical learning and biological understanding can improve predictive skill for breeding applications. Model development and analytical approaches will be iterative as more information is gained through the process development pipeline and new data types are integrated. Closing the breeding–agronomy–production loop has potential to optimize both the effectiveness of the breeding program and farmers’ production. We conclude that plant breeders have the tools to increase both *r*_*a*(MET)_ and *r*_*a*(MET,TPE)_. Considering the sustained creation of drought-tolerant maize, >150 hybrids over a decade, plant breeders can take aim at the mixture of drought-prone and water-sufficient environments within the TPE using a concerted approach to harness CGM–GS technology, gap analyses, and precision phenotyping within MSEs designed to be predictive of the frequent drought-prone environments encountered within the TPE. The increasing availability of sensors, communication systems, and decision support systems will probably contribute to accelerate the application of CGM–GS in breeding to increase genetic gain or close genetic gain yield gaps, and the application of trained CGMs in farmers’ fields to close productivity and sustainability gaps.

## Abbreviations

AQ: AQUAmax® drought-tolerant maize brand
CGM: crop growth model
GS: genomic selection
MET: multienvironment trial
TPE: target population of environments
Δ*r*: prediction accuracy differential
